# The CORTEX Project: A Pre–Post Randomized Controlled Feasibility Trial Evaluating the Efficacy of a Computerized Cognitive Remediation Therapy Program for Adult Inpatients with Anorexia Nervosa

**DOI:** 10.3390/jpm15090430

**Published:** 2025-09-08

**Authors:** Giada Pietrabissa, Davide Maria Cammisuli, Gloria Marchesi, Giada Rapelli, Federico Brusa, Gianluigi Luxardi, Giovanna Celia, Alessandro Chinello, Chiara Cappelletti, Simone Raineri, Luigi Enrico Zappa, Stefania Landi, Francesco Monaco, Ernesta Panarello, Stefania Palermo, Sara Mirone, Francesca Tessitore, Mauro Cozzolino, Leonardo Mendolicchio, Gianluca Castelnuovo

**Affiliations:** 1 Department of Psychology, Catholic University of Milan, 20123 Milano, Italy; gloria.marchesi@unicatt.it (G.M.); giada.rapelli@unicatt.it (G.R.); gianluca.castelnuovo@auxologico.it (G.C.); 2 IRCCS Istituto Auxologico Italiano, Clinical Psychology Research Laboratory, 28824 Piancavallo, Italy; 3 Scuola di Specializzazione di Psicologia Clinica, Università Cattolica del Sacro Cuore, 20123 Milano, Italy; davide.cammisuli1@unicatt.it; 4 IRCCS Istituto Auxologico Italiano, Laboratorio Sperimentale di Ricerche di Neuroscienze Metaboliche, 28824 Piancavallo, Italy; f.brusa@auxologico.it (F.B.); l.mendolicchio@auxologico.it (L.M.); 5 Center for Eating Disorders, AAS n5 ‘Friuli Occidentale’, 33170 Pordenone, Italy; gianluigi.luxardi@gmail.com (G.L.); saramirone1982@gmail.com (S.M.); 6 Department of Psychology and Health Sciences, Pegaso University, 80143 Naples, Italy; giovanna.celia@unipegaso.it; 7 Centro per la Diagnosi e Cura dei Disturbi del Comportamento Alimentare, Casa di Cura Beato Palazzolo, 24122 Bergamo, Italy; alessandro.chinello@gmail.com (A.C.); chiara.cappelletti@casadicurapalazzolo.it (C.C.); simone.raineri@casadicurapalazzolo.it (S.R.); zappaluigi.enrico@tiscali.it (L.E.Z.); 8 Department of Mental Health, Azienda Sanitaria Locale Salerno, 84132 Salerno, Italy; stefanialandi173@gmail.com (S.L.); fmonaco1980@gmail.com (F.M.); ernestapanarello@gmail.com (E.P.); stefaniapalermo94@gmail.com (S.P.); 9 European Biomedical Research Institute of Salerno (EBRIS), 84125 Salerno, Italy; 10 Department of Humanities, Philosophy and Education, University of Salerno, 84084 Fisciano, Italy; ftessitore@unisa.it (F.T.); mcozzolino@unisa.it (M.C.)

**Keywords:** cognitive remediation therapy, anorexia nervosa, randomized controlled trial, cognitive flexibility, motivation to change, clinical psychology

## Abstract

**Background/Objectives:** Anorexia nervosa (AN) is marked by cognitive deficits, particularly reduced mental flexibility and weak central coherence, which may sustain the core psychopathological symptoms. While cognitive remediation therapy (CRT) has shown efficacy in improving these cognitive processes in AN, evidence on computer-based CRT remains limited. This study aims to evaluate the feasibility and efficacy of integrating computer-assisted cognitive remediation therapy (CA-CRT) into standard nutritional rehabilitation (treatment as usual, TAU) to improve the targeted cognitive and psychological parameters among inpatients with AN in a more personalized and scalable way. **Methods**: A multicenter randomized controlled trial (RCT) will be conducted. At least 54 participants with a diagnosis of AN will be recruited at each site and randomized into either the experimental or control group after initial screening. The intervention will last five weeks and include 15 individual CA-CRT sessions alongside 10 individual CR sessions, delivered in addition to standard care. The primary and secondary outcomes will be assessed at the end of the intervention to evaluate the changes in cognitive flexibility, central coherence, and psychological functioning. **Results**: Participants receiving CA-CRT are expected to develop more flexible and integrated thinking styles and achieve greater improvements in clinical outcomes compared to those receiving standard care alone, supporting a more personalized therapeutic approach. **Conclusions**: These findings would underscore the feasibility and clinical value of incorporating CA-CRT into standard inpatient treatment for AN. By specifically targeting cognitive inflexibility and poor central coherence in a scalable, individualized format, CA-CRT may enhance treatment effectiveness and support the development of patient-centered interventions tailored to the cognitive profiles of individuals with AN.

## 1. Introduction

Anorexia nervosa (AN) is a severe and chronic eating disorder characterized by a distorted perception of body size and shape and a fear of gaining weight or being fat, coupled with persistent severe food restriction [1]. It is one of the most common disorders in adolescence, with the highest mortality rates of any mental health condition [2]. The etiology of AN is multifactorial and complex [3,4], and it is often accompanied by psychological, physical, and cognitive complications [5,6]. Therefore, the treatment of AN remains a considerable clinical challenge across the lifespan. In children and adolescents, family-based treatment (FBT) is widely recognized as the first-line intervention, involving active parental participation in refeeding and behavioral restructuring, with robust evidence supporting its effectiveness in promoting weight restoration and reducing eating disorder psychopathology [7,8]. Other evidence-based approaches, such as enhanced cognitive-behavioral therapy (CBT-E), have demonstrated comparable outcomes and represent a viable alternative when FBT is not feasible or acceptable, as well as being a recommended treatment choice in adults [9].

Despite these available options, recent systematic reviews and meta-analyses consistently reported discouraging outcomes, with non-response rates ranging from 30% to 50%, high treatment dropout, and frequent relapse following weight restoration. For example, Berends et al. (2018) reported relapse rates of approximately 31% within 1–2 years [10], while Frostad et al. (2022) found that nearly half of patients relapsed within 12 months post-discharge [11]. A 2024 meta-analysis by de Rijk et al. confirmed a mean relapse rate of 37% over an average follow-up of 31 months, identifying a lower post-treatment BMI and higher baseline depression as significant predictors of relapse [12]. Similarly, a rapid review by Miskovic-Wheatley et al. (2023) reported relapse rates ranging from 41% at the 1-year follow-up to 30% at 22 years [13]. These findings underscore the urgent need for novel, adjunctive treatment strategies that address not only the core maintaining mechanisms of AN—such as weight restoration and disordered eating behaviors—but also broader domains essential for sustained recovery. These include improvements in psychological well-being, interpersonal functioning, emotional regulation, identity development, and overall quality of life, which are often profoundly affected by the disorder. Interventions targeting these broader outcomes should be designed to complement existing treatment frameworks and be feasibly integrated into established care pathways [14,15].

In particular, the consistent findings in the literature highlight two core neurocognitive difficulties commonly associated with AN: cognitive inflexibility—primarily manifested as weak set-shifting abilities [16]—and extreme attention to detail, often described as weak central coherence [17,18,19]. Cognitive flexibility refers to the ability to adjust and respond to evolving environmental requirements by flexibly switching between different rules, tasks, actions, and mental frameworks [20]. Consequently, impaired set-shifting or cognitive inflexibility leads to rigid or persistent thinking and behavior. Central coherence pertains to the ability to process information globally or holistically, whereas weak central coherence is reflected by an excessive focus on detail [21].

In individuals with AN, both impaired set-shifting and diminished central coherence commonly manifest through obsessive preoccupations related to food, body shape, and weight, as well as compulsive behaviors like calorie counting and excessive exercising. These cognitive aspects have been suggested to contribute to poor treatment engagement and response [22]. As a result, they have been included in the maintenance models of AN and targeted by specialized treatment approaches, such as cognitive remediation therapy (CRT) [23,24]. CRT was originally developed for people with brain injuries but has been adapted to help individuals with schizophrenia and, more recently, AN [25].

It is designed to be a brief intervention delivered as an adjunct to traditional interventions for AN [26], and it consists of cognitive exercises focused on the development of new thinking strategies and processes that are not directly related to emotions concerning eating and nutrition (i.e., weight and shape concerns or dietary restrictions).

CRT targets cognitive deficits by focusing on how individuals think rather than on the specific content of their thoughts. Increasing awareness of one’s habitual cognitive style enables the application of CRT exercises to modify maladaptive patterns of daily living—for instance, inflexible adherence to rigid routines that hinder social participation, repetitive checking behaviors that prolong task completion, or disproportionate time allocation to planning meals or exercise regimens at the expense of occupational, recreational, or interpersonal activities [27]. Preliminary research exploring the effectiveness of CRT in persons with AN showed that most find CRT acceptable and demonstrated the efficacy of CRT in improving cognitive flexibility, motivation to change, and better quality of life, with medium to large effect sizes in individuals with AN [28,29,30,31,32,33,34,35,36]. Still, how best to use CRT for the treatment of AN continues to be the subject of investigation.

When applied to individuals with schizophrenia and related disorders, CRT is frequently administered with the assistance of a computer-based platform, a type of treatment delivery referred to as computer-assisted cognitive remediation therapy (CA-CRT) [37,38,39,40]. It involves the completion by patients of a series of tasks on a computer while a trained facilitator engages them in a discussion about the tasks. Patients are encouraged to verbalize their process and to perform self-check-ins to gauge their performance.

The computer-assisted delivery format offers several theoretical and practical advantages in terms of psychological interventions, including improved scalability, potential for personalization of task difficulty and content, and enhanced patient engagement through interactive features. Moreover, digital platforms can facilitate remote delivery, making interventions more accessible to individuals who face geographic, mobility, or scheduling barriers. These benefits align with the growing interest in digital mental health tools as a complement or alternative to traditional face-to-face interventions [41,42,43].

While research on digital interventions for eating disorders remains limited, emerging findings suggest that computer- or internet-delivered programs may be effective in reducing core symptoms, improving motivation to change, and increasing access to treatment. However, most of the existing evidence focuses on guided self-help CBT for bulimia nervosa or binge eating disorder, and—to date—we are unaware of any research focused on testing digital adaptations of CRT for AN. The only study on the topic aimed at detecting changes in brain function potentially associated with CRT in AN [44], which led to encouraging results.

Therefore, the present multicenter randomized controlled trial (RCT) aims to implement and evaluate a personalized CA-CRT program for individuals with AN as an adjunct to standard nutritional rehabilitation (treatment as usual, TAU). The study will test the feasibility and efficacy of CA-CRT in enhancing selected neuropsychological outcomes (cognitive flexibility and central coherence) and psychological measures (eating disorder symptomatology, health-related quality of life, and motivation to change) among inpatients with AN. The feasibility of the study will be evaluated through indices such as the recruitment and retention rates and the patient outcomes. These metrics will help determine not only whether the intervention is acceptable but also whether it can be implemented in routine clinical settings. By delivering a structured, scalable, and individually tailored intervention, the trial seeks to promote more effective, patient-centered care approaches. It is hypothesized that participants allocated to the experimental group will demonstrate greater improvements in the targeted outcomes compared to those receiving standard care alone over the 5-week intervention period. Specifically, the primary hypothesis is that participants in the CA-CRT group will show significantly greater improvement from baseline to post-treatment than those in the TAU group in relation to cognitive flexibility, as measured by the Wisconsin Card Sorting Test (WCST), the Phonemic/Semantic Alternate Fluency Test, the Trail Making Test (TMT), the Test of Attentional Performance (TAP), the Detail and Flexibility Questionnaire (DFlex), and the Cognitive Flexibility Inventory (CFI). Improvements are also expected in the Central Coherence Index (CCI) of the Rey–Osterrieth Complex Figure (ROCF-C).

The secondary hypothesis is that the CA-CRT group will show greater reductions in their eating disorder symptoms (EDE-Q), as well as greater improvements in their quality of life (WHOQOL-BREF) and readiness to change (URICA), compared to those receiving TAU.

## 2. Materials and Methods

The study will be conducted as a multicenter randomized controlled trial across three specialized Italian facilities dedicated to the nutritional rehabilitation and treatment of those with eating and weight disorders: IRCCS Istituto Auxologico Italiano in Piancavallo Oggebbio (VB), Beato Palazzolo Hospital in Bergamo, and the Mariconda Regional Eating Disorders Unit in Salerno.

Participants who meet the inclusion criteria will be enrolled on a rolling basis and randomized in a 1:1 ratio into two groups using a secure, computerized, web-based system. The experimental group will receive the CA-CRT intervention in addition to the standard rehabilitation program (treatment as usual, TAU), while the control group will follow TAU alone.

In line with national guidelines, the standard TAU protocol will be applied consistently across all the participating sites [45].

The study was registered on ClinicalTrials.gov NCT05912036 (https://clinicaltrials.gov/study/NCT05912036) on 20 June 2023 and follows the SPIRIT Statement for reporting a randomized trial protocol. The corresponding SPIRIT checklist is included as a supplementary document (see Supplementary Table S1).

### 2.1. Ethical Statement

All the procedures performed in the study will be run following the ethical standards of the institutional and/or national research committee and in compliance with the Declaration of Helsinki and its later amendments or comparable ethical standards.

The study was approved by the Ethical Committee of IRCCS, Istituto Auxologico Italiano, (ID: 03C214) on 17 May 2022. Important protocol amendments will be communicated promptly to investigators, ethics committees, and regulatory authorities as required.

### 2.2. Study Population

Participants will be recruited in the first week of admission to the 1-month nutritional rehabilitation program in the three specialized centers. The inclusion criteria will be as follows: (1) receiving a diagnosis of AN according to the DSM-5 criteria [1]; (2) being over 18 years old; (3) having fluent Italian; and (4) signing the informed consent to participate in the study. Participants will be excluded from the study if they present with (1) specific learning disabilities (SLDs) or intellectual disability, (2) psychosis, (3) head injury, (4) history of psychotropic substance use, or (5) other clinical sensorial impairment (visual or hearing one) preventing them from following the intervention. Participants receiving pharmacological treatment will not be excluded from the study.

### 2.3. Sample Size Calculation

The minimum sample size required for this study has been computed using an *a priori* sample size calculator (G*Power 3.1.9.2 software) for the *F* tests [46,47,48]. Participants will be randomly allocated into two groups: (A) TAU plus CA-CRT condition (experimental group) and (B) TAU-only condition (control group). Moreover, the recruits will be assessed before and after the intervention (i.e., 5 weeks). The Pillai V has been set *a priori* to take a value of 0.2, with an effect size of 0.5. Moreover, the type I error (*α*) rate has been set at 0.05 (two-sided), and the power (1 − *β*) at 0.95, respectively. The results showed that there is a 95% chance of correctly rejecting the null hypothesis of no significant interaction effect, with 54 recruits in total (27 per group) for each center.

### 2.4. Randomization and Blinding

**Figure 1:**
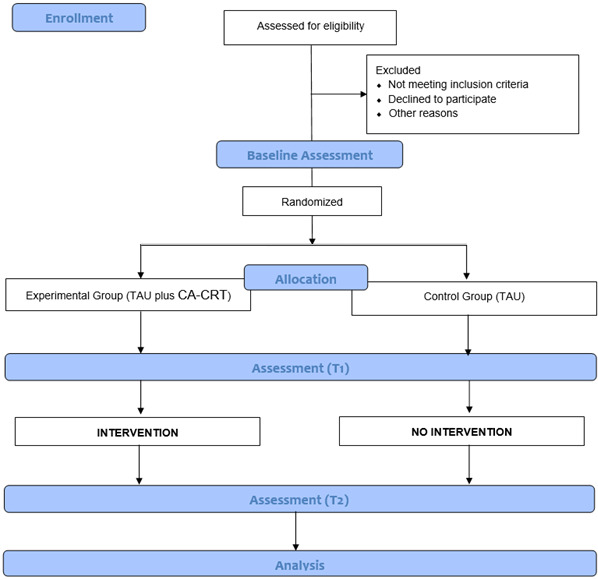
Flowchart of the CORTEX study.

Randomization will be stratified by center and performed using the permuted block technique within each site by a researcher independent of the study. Due to the nature of the intervention, the group allocation cannot be concealed from the participants or the provider of the intervention. Participants will be assigned to one of two conditions within the first week from the baseline assessment (Figure 1).

### 2.5. Measures

Demographic (age, gender, education, civil status, job status) and clinical information (age at disease onset, body mass index—BMI—at admission to the clinics, pharmacological treatment, and psychiatric comorbidities) will be collected from each participant’s medical record at baseline.

The Italian versions of the following psychodiagnostic measures will be collected at baseline for an in-depth evaluation of the psychological profile of the participants.

The Beck Anxiety Inventory (BAI) [49,50]: A self-report questionnaire consisting of 21 items assessing the severity of anxiety-related psychological and cognitive symptoms (e.g., sweating, tingling, trembling hands, fear that the worst might happen). Each item is rated on a 4-point Likert scale, ranging from 0 to 3, with higher scores indicating elevated levels of anxiety. The total score is calculated by summing the score for all the items and can be interpreted as follows: 0–7 = minimal anxiety; 16–25 = mild anxiety; 26–63 = severe anxiety.

The Beck Depression Inventory-II (BDI-II) [50,51]: A self-report questionnaire consisting of 21 items assessing the severity of depressive symptoms. It allows for evaluation of two distinct factors, i.e., the Somatic–Affective dimension, on the somatic–affective manifestations of depression (e.g., loss of interest, loss of energy, changes in sleep and appetite, agitation, and crying), and the Cognitive factor, about personal beliefs on depression (e.g., pessimism, guilt, self-criticism, and self-esteem). Each item is rated on a 4-point Likert scale ranging from 0 to 3, with higher scores indicating elevated levels of depression. The total score is calculated by summing the score for all the items and interpreted as follows: 0–13 = minimal depression; 14–19 = mild depression; 20–28 = moderate depression; 29–63 = severe depression.

The Obsessive and Compulsive Inventory-Revised (OCI-R) [52,53]: A self-report questionnaire consisting of 18 items loaded onto six different subscales: (1) Washing, (2) Obsessing, (3) Hoarding, (4) Ordering, (5) Checking, and (6) Mental Neutralizing. Each item rates the individuals’ distress on a 5-point Likert scale, ranging from 0 (not at all) to 4 (very much).

Moreover, the Standard Progressive Matrices (SPM) [54,55] will be administered. It consists of 60 items measuring nonverbal intelligence divided into 5 sheets (12 items each). Each item requires completion of a series of figures with a missing part that is presented among distractors, according to an increasing difficulty criterion.

Then, the Italian versions of the following neuropsychological tests will be collected pre- and post-treatment and used as the primary outcomes.

The Wisconsin Card Sorting Test (WCST) [56,57] will be used to assess abstract reasoning, mental flexibility, and problem-solving. The examinee has to match stimulus cards to one of four category cards. Cards can be matched by shape (cross, circle, triangle, star), color (red, blue, yellow, green), and number (one, two, three, four). The rule must be worked out by the feedback provided by the examiner (“right” or “wrong” response). When a rule is identified (i.e., 10 consecutive correct matches), the examiner changes the criterion without saying anything, and the examinee has to identify the new rule. Scoring provides the following indices: total mistakes, total correct responses, completed categories, perseverative mistakes, non-perseverative mistakes, percentages of perseverative mistakes, attempts to complete the first category, inability to retain the criterion, and task learning.

The Phonemic/Semantic Alternate Fluency Test [58] will be used to assess the verbal set-shifting ability. The examinee has to produce word pairs unusually, starting from a phonemic cue (letter A, F, S) and a semantic cue (color, animal, fruit), in 60 s. The raw score (number of word pairs obtained for each association (A–color; F–animal; S–fruit)) is adjusted for age, education, and gender; thus, it is converted into an equivalent score. Moreover, a composite shift index evaluating working memory is determined as follows: Total Alternate Fluencies/(Total Phonemic Fluencies + Total Semantic Fluencies/2).

The Trail Making Test (TMT) [59] is a measure of visual processing speed (Part A) and dual-task/cognitive flexibility (Part B). In the first part, the examinee is required to sequentially join a series of numbered circles and, in the second part, to alternate sequences of numbers and sequences of letters by linking the stimuli together (e.g., 1-A → 2-B → 3-C). Scoring is based on the time completion for each part. The raw score is adjusted for age and education; thus, it is converted into an equivalent score.

The Rey–Osterrieth Complex Figure (ROCF-C) [60] is a pen-and-paper task measuring global processing ability. Subjects are required to copy a complex figure (ROCF-C).

A Central Coherence Index (CCI) is derived from the composite score of two independent indices: the order of construction index (order in which the elements of the figure are drawn) and the style index (continuity of drawing). The CCI ranges from 0 (most detailed approach) to 2 (most coherent approach), where higher scores mean a more coherent drawing style [61].

The Test of Attentional Performance (TAP) [62,63] is a computerized battery assessing various attentional domains. The following subtests will be administered.

- Sustained attention (15 min): A series of stimuli are displayed on the screen, and the examinee has to detect the target among the distractors during a prolonged presentation. The stimuli differ in dimension, color, shape, size, and filling.
- Cross-modal integration (2 min and 50 s): Participants need to identify the crucial combination of a preceding tone (i.e., high or low) with a subsequent visual stimulus (i.e., an arrow pointing up or down).
- Flexibility, from a simple (1 min and 45 s) to a complex (3 min) configuration: This subtest involves a set-shifting task requiring participants to react to complementary target stimuli (e.g., a letter and a number) in an alternating manner.
- Inconsistency (3 min): This test examines the sensitivity to interference (i.e., stimulus-response incompatibility). The subject is required to respond with the right or left hand, regardless of the direction signaled by the stimulus.
- Working memory (5 min): This test examines the management of information flow and the updating of data by detecting differences in number sequences presented in series.

Each subtest is preceded by a run-in, and the difficulty level of the task may change, according to the examiner’s guide. The TAP subtests are measured by the reaction times and accuracy determined as correct responses, false responses, omissions, and anticipations (i.e., responses within 100 ms).

The Detail and Flexibility Questionnaire (DFlex) [64,65]: A self-report questionnaire consisting of 24 items on a 6-point Likert scale (from 1 = strongly disagree to 6 = strongly agree) and assessing cognitive rigidity (RC, 12 items) and attention to detail (AD, 12 items). The subscale scores range from 12 to 72 and are calculated by summing the RC items and the AD items.

The Cognitive Flexibility Inventory (CFI) [66,67]: A self-report questionnaire consisting of 20 items used to assess two different subscales: the Alternative subscale, which evaluates the ability to perceive multiple alternative explanations for life events and human behavior, and the ability to generate multiple alternative solutions to difficult situations; and the Control subscale, which assesses the tendency to perceive difficult situations as controllable. Each item is scored on a 7-point Likert scale (from 1 = strongly disagree to 7 = strongly agree). The scoring requires inverse scoring of the selected items. The total score is calculated by the sum of all the items. Higher scores indicate greater cognitive adaptability and flexibility to be used in stressful situations; lower scores indicate greater cognitive rigidity associated with less cognitive flexibility in stressful situations.

The Italian versions of the following psychological measures will be also collected pre/post-treatment and used as secondary outcomes.

The Eating Disorder Examination Questionnaire (EDE-Q) [68,69] is a self-report questionnaire consisting of 33 mixed-type items (yes/no, 7-point Likert scales and visual analog scales) assessing the core symptoms of the eating disorder in the past 28 days. The EDE-Q has four subscales (i.e., Restraint, Eating Concern, Shape Concern, and Weight Concern) and a global score is calculated by the mean of all four subscales. Higher scores indicate an elevated frequency or clinical severity of eating disorder symptoms.

The World Health Organization Quality of Life (WHOQOL-BREF) [70]: A self-assessment questionnaire consisting of 26 items rated on a 5-point Likert scale and grouped into four dimensions: physical health (i.e., mobility, daily activities, functional capacity, energy, pain, and sleep), psychological health (i.e., self-image, negative thoughts, positive attitudes, self-esteem, mindset, learning ability, memory concentration, religion, and mental condition), social relationships (i.e., personal relationships, social support, and sex life) and environmental health (i.e., financial resources, safety, health and social services). It also contains QoL and general health items. The score ranges from 25 to 130 and is transformed linearly into a 0–100 scale.

The University of Rhode Island Change Assessment Scale (URICA) [71]: A self-report questionnaire consisting of 28 items rated on a 5-point Likert scale (from 1 = strongly disagree to 5 = strongly agree) representing four primary stages of change in Prochaska and DiClemente’s Trans-Theoretical Model (1982) [71], with 7 items for each of the following subscales: Precontemplation, Contemplation, Action, and Maintenance. To obtain a Readiness to Change total score, the items from each subscale must be summed, then divided by 7 to obtain the mean for each subscale. Following this, the means for the Contemplation, Action, and Maintenance subscales must be summed, and the Precontemplation mean subtracted (C + A + M − PC = Readiness).

Psychodiagnostic and psychological measures will be collected in group settings, in a dedicated room, by clinical psychologists independent of the study. Neuropsychological tests will be administered individually by neuropsychologists independent of the study in appropriate settings.

The duration of the assessment will be two hours for each patient, divided into two sessions (group and individual) on consecutive days.

Personal information will be entered and coded securely with quality control procedures (e.g., double data entry, range checks).

Data will be shared securely in compliance with data protection regulations to maintain participant confidentiality before, during, and after the trial.

### 2.6. Procedure

Patients with AN will be invited to take part in the study at admission to the rehabilitation centers, according to the above-mentioned inclusion/exclusion criteria.

Before the randomization, they will be asked to sign the informed consent to participate in the study and to complete the psychodiagnostic (BAI, BDI II, OCI-R) measures.

Randomization will then be stratified by center and performed using the permuted block technique within each site by a researcher independent of the study. Participants will be assigned to one of two conditions: both groups will receive the usual hospital care (TAU), and the experimental group, in addition, will receive the CA-CRT treatment.

Before starting the program, all the participants will undergo neuropsychological testing (primary measures) and complete self-report psychological questionnaires (secondary measures).

The standard nutritional rehabilitation program is set up as a multidisciplinary treatment approach merging group therapies, individual therapies, and dietetic management in line with national guidelines [45]. Dietetic rehabilitation aims to encourage a change in eating habits and includes mealtime supervision, as well as both individual and group counseling provided by dietitians.

Individual sessions last from 30 to 45 min and encompass dietary assessment, evaluation of nutrient intake and adequacy, nutritional and anthropometric status, eating patterns, weight history, and patients’ readiness to change. Group meetings of around 20 people are provided every week and last about one hour. The main topics discussed include biological hunger, satiety, nutrients, weight management, and behavior change strategies for preventing relapse.

The patients’ meal plan is determined by the dietitian to gain half a kilogram (one pound) per week.

Meals last 60 min, followed by a 30 min mandatory resting period, where patients and staff members are together in a dedicated room. There are staff members present at all times during the meal and resting period.

Psychotherapy is provided once a week by a licensed psychotherapist in an individual setting and lasts about 45 min. Further, 1 h psychoeducational groups led by educationalists, psychologists–psychotherapists, or psychiatrists and focused on the relationship between food and emotions are provided weekly.

Bodily experience is also specifically targeted through body image therapy (provided twice a week for 30 min by physiotherapists) and group dance therapy (provided once weekly by a psychologist and lasting 1 h) to help patients cope with a distorted body image.

Lastly, low-intensity physical activity based on gentle gymnastic sessions (3 times per week, 30 min per session) is provided to treat and normalize patients’ views and attitudes toward physical activity. Tailored activities in both individual and group settings are planned upon physical and functional assessment by professional physiotherapists.

The CA-CRT treatment will entail cognitive exercises aimed at improving more adaptive thinking styles, encouraging patients’ reflection on their way of elaborating information, and exploring the possibility of implementing new schemas in everyday life. The CA-CRT program will employ a wide range of cognitive tasks to address cognitive inflexibility and weak central coherence using the Italian translation of the CRT original manual [72]. The combined treatment will consist of 10 individual 40 min CA-CRT sessions, 2 times a week for 5 weeks, plus 15 individual CA-CRT 30 min sessions, 3 times a week for 5 weeks.

The description of the CA-CRT exercises for each session is reported in Table 1. The CA-CRT will consist of executive functions tasks, i.e., “sequence of symbols” and “answers by category”, as well as visual–perceptual processing, i.e., “figure memory”, and “geometric figure grids” retrieved from the Erica^®^ software (Giunti O.S., Florence, Italy). 

The total duration of the research will be 6 months, considering a discontinuous enrollment related to the patients’ admission to the clinics.

At the end of the treatment (after 5 weeks), recruited participants in both conditions will be administered the neuropsychological tests and the psychological questionnaires again.

Adherence will be supported through participant education and monitored via scheduled assessments and treatment logs.

As the study will be conducted in an inpatient unit with close clinical supervision, a formal monitoring plan is not required.

The experiential treatment can be discontinued in response to adverse events, participant request, or clinical changes. Participants will receive appropriate medical care for any trial-related harm.

### 2.7. Statistical Analysis

Statistical analyses will be performed using SPSS version. 24.0 [73]. Descriptive analyses will be conducted on the experimental group and control group. After preliminary statistical assumption satisfaction, an independent samples T-test will be used to determine whether the groups differ in the psychodiagnostic measures administered at baseline. These potential differences will be used as covariates in the model. A multivariate analysis of covariance (MANCOVA) will then be conducted (factors: experimental group, control group, time; dependent variables: scores obtained in the neuropsychological and psychological measures; covariates: scores obtained in the psychodiagnostic measures at baseline). We will adopt this design because the dependent variables concerning the executive functions (WCST, alternate fluency, and TMT), as well as visuo-perceptive processing (ROCF-C), are moderately but significantly associated, given their involvement in frontal lobe abilities.

Partial eta-squared will be used to determine the effect size of the intervention. The analysis will be performed by an independent statistician, blinded to the treatment allocation, who will discuss the results with the researchers. Missing data will be handled using appropriate statistical methods to minimize bias and ensure robust results.

## 3. Expected Results

To our knowledge, this will be the first RCT aimed at evaluating the feasibility and efficacy of CA-CRT in inpatients with AN undergoing nutritional rehabilitation. By delivering an individualized, structured intervention targeting specific cognitive deficits, this study aims to address the inconsistent conclusions highlighted in prior meta-analytic work [23]. It is expected that participants in the experimental group will demonstrate greater improvements in the neuropsychological outcomes—including cognitive flexibility, verbal fluency, visuospatial processing, and attention—as well as the psychological measures such as eating disorder symptomatology, health-related quality of life, and motivation to change. Participants are anticipated to develop a more flexible and integrative thinking style tailored to their unique cognitive profiles. Additionally, conducting the study across multiple specialized centers will enhance the generalizability and external validity of the findings, supporting the broader implementation of personalized, scalable therapeutic approaches for AN.

Still, several potential limitations should be considered in terms of the present study design. First, although the randomized controlled design strengthens the internal validity, recruitment will be conducted in a limited number of specialized clinical centers, which may reduce the generalizability of the findings to other settings or the broader population of individuals with AN. Second, the study will rely on a pre–post assessment with no intermediate follow-up; as a result, it will not be possible to evaluate the persistence of the treatment effects over time. Third, the inclusion of multiple neuropsychological and psychological measures increases the comprehensiveness of the assessment but also raises the potential for type I errors. Fourth, the intervention will be delivered in addition to standard rehabilitation treatment, which could vary between participants and centers, introducing heterogeneity that may influence the outcomes. Last, for ethical reasons, it will not be possible to control for participants’ pharmacological treatment or to restrict access to other forms of psychotherapy before or during the trial; while these factors will be recorded, they may introduce variability in the treatment response.

## 4. Conclusions

Eating disorders represent a significant public health challenge given their rising prevalence [74,75]. There is an urgent need for targeted interventions that address the cognitive mechanisms underlying psychopathology in a personalized manner. By equipping patients with tailored, evidence-based strategies to improve their thinking styles and self-management skills, CA-CRT holds promise as an effective adjunctive treatment that can be routinely integrated into inpatient and outpatient settings. Such approaches may not only enhance individual outcomes but also contribute to more sustainable and cost-effective healthcare delivery [76], aligning with the goals of personalized and precision mental healthcare.

## Figures and Tables

**Table 1:** Description of the CA-CRT exercises.

Title	Content
Introductory script	This is an introduction to explain to the examinee the idea of what CRT is and what he/she is expected to accomplish by its practice.
Complex pictures	The exercise consists of describing a complex figure to the therapist so that he/she can draw it; then the examinee is asked to reflect on the task and on what he/she thinks about it.
Main idea task	The exercise consists of reading a letter or a text and trying to summarize it in a few sentences.
Illusions task	The exercise consists of observing an image and describing it.
Stroop material	For each of the 7 Stroop exercises, the idea is to encourage the subject to be rapid and fast and to increase attentional shifts.
Switching attention task	To practice switching between two different names (e.g., animal and cities) by holding in mind the English alphabet letter progression (e.g., A alligator; B Beijing; C camel).
Embedded words task	The exercise consists of giving the subject a sheet with some words and asking him/her to follow the instructions at the beginning of the page.
Word search task	The exercise consists of finding the words listed between the letters on the sheet.
Estimating task	The exercise consists of marking the center of some figures; the mark does not have to be perfectly in the center, but a good estimate is necessary.
Up and down task	This exercise consists of training the ability to shift attention from one rule to another one.
Card stack	A deck of cards is necessary for this task; the aim is to sort the cards following rules based on color, shape, or number.
Maps task	The exercise consists of choosing one of the presented maps and then observing and describing them using different ways.
Prioritizing task	The exercise consists of encouraging the examinee to plan something according to priorities.
Bigger picture task	The exercise consists of writing a description of an image to be compared with the therapist’s description of the same image.
How to plant a sunflower	The exercise consists of describing how to do something; the aim is to help the examinee think in a general way.
Search and count	First, this exercise consists of observing all the symbols in a sheet and deleting the circles; after some time, the examinee is required to count from 1 to 20 simultaneously; after that, the examinee is asked to stop deleting circles and start deleting triangles, always counting from 1 to 20.
Switching time zones	The exercise consists of answering some questions about time zone maps.

## Data Availability

No new data were created or analyzed in this study. The original contributions presented in this study are included in the article. Further inquiries can be directed to the corresponding author.

## Supplementary Materials

The following supporting information can be downloaded at https://www.mdpi.com/article/10.3390/jpm15090430/s1, Table S1: SPIRIT 2025 checklist.
